# Rehabilitation participation and one‐year cognitive and affective changes in adult day service users with dementia

**DOI:** 10.1002/alz.088578

**Published:** 2025-01-09

**Authors:** Naofumi Tanaka, Yoshitaka Ouchi, Yin‐Jung Wang, Masashi Kasuya

**Affiliations:** ^1^ Saitama Medical University International Medical Center, Hidaka, Saitama Japan; ^2^ Medical corporation Jinsenkai, Sendai, Miyagi Japan; ^3^ Tohoku Fukushi University, Sendai, Miyagi Japan; ^4^ Miyagi University, Daiwa, Miyagi Japan

## Abstract

**Background:**

Day care services provide various activities, including rehabilitation, to maintain and improve physical function and social participation of older people. However, the relationship between rehabilitation participation and emotional changes in older adults with dementia who use day care services is not well understood.

**Method:**

The participants were 157 older adults living in the community who used day‐care services. Users with MMSE scores less than 10 at the initial and one‐year evaluation were excluded. Other assessments were the Geriatric depression scale‐15 (GDS‐15), the Apathy evaluation scale (AES) and the Short physical performance battery (SPPB). Rehabilitation participation was averaged over three consecutive days of use using the Pittsburgh Rehabilitation Participation Scale (PRPS), with the group with a PRPS greater than 4 points divided into two groups: good and poor rehabilitation participation groups. MMSE, GDS, AES and SPPB scores were compared using Wilcoxon’s rank sum test at the initial and one‐year follow‐up.

**Result:**

The age of the participants was 83 ± 7 years and 43/114 were men/women. When further divided into three groups according to MMSE score (≥24, 20‐23 and 19‐10), the good rehabilitation group with an MMSE score of 19‐10 had a significantly higher AES score (p<0.001) and a significantly lower SPPB score (p = 0.016).

**Conclusion:**

Among older adults with dementia using day care services, the group with good participation in rehabilitation was suggested to be at risk of increased apathy and reduced lower limb motor function after one year.

